# MER41 Repeat Sequences Contain Inducible STAT1 Binding Sites

**DOI:** 10.1371/journal.pone.0011425

**Published:** 2010-07-06

**Authors:** Christoph D. Schmid, Philipp Bucher

**Affiliations:** 1 Swiss Institute of Bioinformatics, EPFL (École Polytechnique Fédérale de Lausanne) SV ISREC (The Swiss Institute for Experimental Cancer Research) GR-BUCHER, Lausanne, Switzerland; 2 Swiss Tropical and Public Health Institute (Swiss TPH), Basel, Switzerland; 3 University of Basel, Basel, Switzerland; Genome Institute of Singapore, Singapore

## Abstract

Chromatin immunoprecipitation combined with massively parallel sequencing methods (ChIP-seq) is becoming the standard approach to study interactions of transcription factors (TF) with genomic sequences. At the example of public STAT1 ChIP-seq data sets, we present novel approaches for the interpretation of ChIP-seq data.

We compare recently developed approaches to determine STAT1 binding sites from ChIP-seq data. Assessing the content of the established consensus sequence for STAT1 binding sites, we find that the usage of “negative control” ChIP-seq data fails to provide substantial advantages. We derive a single refined probabilistic model of STAT1 binding sequences from these ChIP-seq data. Contrary to previous claims, we find no evidence that STAT1 binds to multiple distinct motifs upon interferon-gamma stimulation *in vivo*. While a large majority of genomic sites with high ChIP-seq signal is associated with a nucleotide sequence ressembling a STAT1 binding site, only a very small subset of the over 5 million potential STAT1 binding sites in the human genome is covered by ChIP-seq data. Furthermore a surprisingly large fraction of the ChIP-seq signal (5%) is absorbed by a small family of repetitive sequences (MER41).

The observation of the binding of activated STAT1 protein to a specific repetitive element bolsters similar reports concerning p53 and other TFs, and strengthens the notion of an involvement of repeats in gene regulation. Incidentally MER41 are specific to primates, consequently, regulatory mechanisms in the IFN-STAT pathway might fundamentally differ between primates and rodents.

On a methodological aspect, the presence of large numbers of nearly identical binding sites in repetitive sequences may lead to wrong conclusions about intrinsic binding preferences of TF as illustrated by the spacing analysis STAT1 tandem motifs. Therefore, ChIP-seq data should be analyzed independently within repetitive and non-repetitive sequences.

## Introduction

The precise spatial and temporal regulation of gene expression remains poorly understood despite an increasing number of species with nearly complete genome sequences available. Proteins which regulate the expression of genes by binding to specific DNA sequences in the vicinity of their targets have been termed transcription factors (TFs). The nucleotide sequences of observed binding sites generally display a considerable variation, which may cause difficulties in the description of binding preferences using sequence motifs. Recent benchmarking studies confirmed that transcription factor binding sites (TFBS) prediction based on statistical motif discovery approaches is unreliable and thus remains a major bottleneck in the study of transcriptional regulatory regions [1], [2]. Identification of regulatory elements based on evolutionary conservation, also known as phylogenetic footprinting, has the evident drawback of missing regulatory elements responsible for diversity among species. Therefore laboratory experiments assaying DNA-protein interactions in vivo remain indispensable. Especially massively parallel sequencing technologies in combination with chromatin immunoprecipitation (ChIP-seq) has proven a very powerful method to locate precisely the DNA elements that physically interact with the targeted protein in the specific cell population [3]. The enhanced precision in the large-scale mapping of binding sites occupied in vivo might also overcome some of the limitations of descriptors of binding sites based on conventional ChIP-chip approaches.

Methods for interpreting ChIP-seq data are currently under intensive development. A common aspect of emerging solutions includes the possibility to recognize and process separately sequence reads from the + and the − strand of pulled down fragments [4]. The exact mapping of the ChIP fragment ends allows consequently for more accurate delineation of DNA regions that interact with the targeted protein. The other main task in the analysis of ChIP-seq data consists in the separation of ‘true signal’ from spurious background associated with ChIP or additional still uncharacterized experimental artifacts. Various approaches of ‘peak calling’ have been applied to determine loci with a clearly increased coverage of ChIP-seq reads. Some of these methods include complex statistical approaches and data from ‘negative control’ experiments [5], [6], [7], [8], [9], [10], [11], [12].

STAT1 is a member of an intensively studied family of TFs with implications in the regulation of immune responses. In resting cells STAT1 is mainly located in the cytosol. Upon stimulation with the cytokine interferon gamma (IFN-γ), STAT1 is trans-located to the nucleus to bind to target DNA sequences. Early studies on relatively few binding sequences defined a consensus sequence TCCNNNGAA of the IFN-γ-activated site (GAS [13]). Subsequent studies established a descriptor of STAT1 binding preferences with an improved specificity by defining a position-specific weight matrix (PWM) derived from the in vitro binding of STAT1 to synthetic oligonucleotides [14]. Described alternative STAT1 binding sites include the ISRE motif [15] and an additional variant of the GAS motif (M2, [7]).

Repetitive sequence elements constitute almost half of the human genome, however their potential functions are still poorly defined. A number of recent studies either predict TFBS within repeat sequences [16], [17], or present evidence for an interaction of regulatory proteins with repeats [18], [19], [20], [21]. In addition to an enhanced positional resolution, ChIP-seq is more effective in mapping DNA-protein complexes located inside repetitive elements, where the ChIP-chip approach faces serious limitations due to cross-hybridization.

The present re-analysis of ChIP-seq data extends the analysis of STAT1 binding for the first time to repetitive sequences. We furthermore show that ChIP-Seq data can be used to analyze the relation of in vivo interactions and extended sequence features such as spacing within regulatory modules of binding sites. A large number of virtually identical binding sites within repeats potentially induces a strong bias in a corresponding binding site model and could lead to wrong conclusions regarding preferential associations and constraints in distances to other TFBS. We present therefore approaches to analyze ChIP-seq data which also take into account the emerging roles of repeats in the regulation of gene expression and evolutionary aspects. All results shown in this article are based on public ChIP-Seq data described in [3] defining the genome-wide distribution of STAT1 protein in HeLa cells upon γ-interferon stimulation.

## Results

### ChIP-peak calling and comparison to other algorithms

ChIP-peak (see methods), defines a set of 4446 STAT1 binding sites highly occupied in IFN-γ-stimulated HeLa cells (list as Files S1 and S2). In accordance with limited amounts of STAT1 in the nucleus of unstimulated cells [13], we obtain only 356 sites if applying peak detection with identical parameters on the control ChIP-seq data set derived from unstimulated HeLa cells. 286 (80%) of these ‘unstimulated sites’ are located within +/- 100bp to a corresponding peak in the stimulated set.

**Figure 1:**
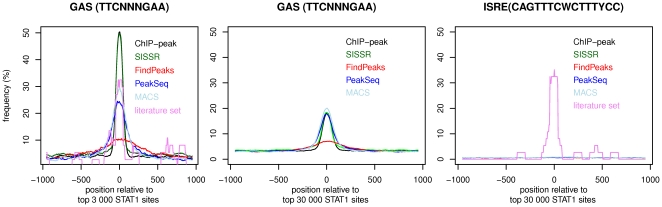
Content of binding sites to assess STAT1 ChIP-seq sets. The frequencies of binding sequences TCCNNNGAA in 4 sets of genomic loci derived from STAT1 ChIP-seq data. ‘ChIP_peak’ contains STAT1 sites as defined in this study, FindPeaks (Robertson et al.), SISSR (Jothi et al.), and MACS (Zhang et al.) are derived of analyses of the identical ChIP-seq data set and PeakSeq (Rozowski et al.) uses an independent STAT1 ChIP-seq data set. The sets contain the top 3000 (left panel) or top 30 000 sites (center & right panel) of the corresponding data. The literature set consists of 37 STAT1 sites collected in the ORegAnno database. The occurrence of GAS is assessed in 100 bp sequence windows (SSA web server http://www.isrec.isb-sib.ch/ssa/). Plotted points represent the center positions of windows relative to the position of the putative STAT1 site predicted from ChIP-seq data. The right panel displays the frequency of the ISRE PWM (Transfac entry M00258).

Next, we aim to compare the results of our peak detection approach to previously described STAT1 sites (or center positions of ‘binding regions’) derived from the identical ChIP-seq data set [3], [7] and to an additional independent STAT1 ChIP-seq data set [12]. The consensus sequence TCCNNNGAA of the IFN-γ-activated site (GAS [13]) does not comprehensively describe all binding sequences of STAT1, but it allows an unbiased assessment of the positional precision of the determination of STAT1 binding in sequence sets. The original analysis of the ChIP-seq data set [3] produced 41582 binding regions with an average size of about 1kb. In our approach the concentration of the counts of ChIP-seq reads at the putative center position of IP fragments considerably enhances the precision and the sensitivity in the detection of sites with high ChIP-seq signal (peaks). We sort the sets of STAT1 binding sites by decreasing confidence levels either according to the coverage by ChIP-seq reads or according to statistical scores provided by the analysis [12]. Comparing equally sized samples of all sets, a significant increase in the average content of GAS consensus sequences precisely at the position of the inferred STAT1 binding sites is consistently observed for all sets (Fig. 1, background content at distant positions <5%). For the 3000 top ranking sites, our ChIP-peak set stands out with the highest and best focused enrichment featuring a GAS within 100bp of almost 50% of the inferred sites. This frequency exceeds that of 37 STAT1 binding sites collected from descriptions in the literature [22]. This apparent discrepancy is most likely explained by a considerable fraction of sites with an alternative STAT1 binding motif (ISRE [15]) in the literature set, while ISRE is not detected in any of the ChIP-seq derived sets (Fig. 1). In the comparison in Fig. 1 the set by Jothi et al. closely follows our set and the algorithm by Rozowsky et al. catches up in the larger sets of 30000 highest ranking sites. These larger sets include also lower affinity sites and accordingly display a lower frequency of ‘perfect’ GAS without mismatches. At least for sites with moderate to high ChIP-seq coverage, the inclusion of information from ‘control’ ChIP-seq data sets in the peak calling [7], [12] (e.g. DNA from unstimulated cells, or from ChIP input, respectively) does not to provide significant advantages in terms of precision and content of consensus STAT1 binding sequences. A sequence motif closely resembling the GAS consensus is associated with large parts of the observed ChIP-seq signal. Allowing one mismatch in the GAS consensus raises its frequency to 95% within 100bp to the determined binding sites in our set. Consensus sequences with mismatches are however unspecific descriptors of binding sites as reflected by background frequencies of 60% in this example.

### Refinement of description of STAT1 binding motif (GAS)

Position-specific scoring approaches using weight matrices (PWM) or Hidden Markow Models (HMM) feature superior performances as descriptors of binding preferences [23]. PWMs enable also the prediction of potential binding sites in genomic sequences. Previous approaches characterized the binding of STAT1 in vitro to synthetic oligonucleotides [14]. We observed that a number of thereby predicted genomic binding sites remain devoid of the now available STAT1 ChIP-seq data [3]. This prompted us to investigate potential differences in characteristics of binding sites in vitro vs. in vivo.

**Figure 2:**
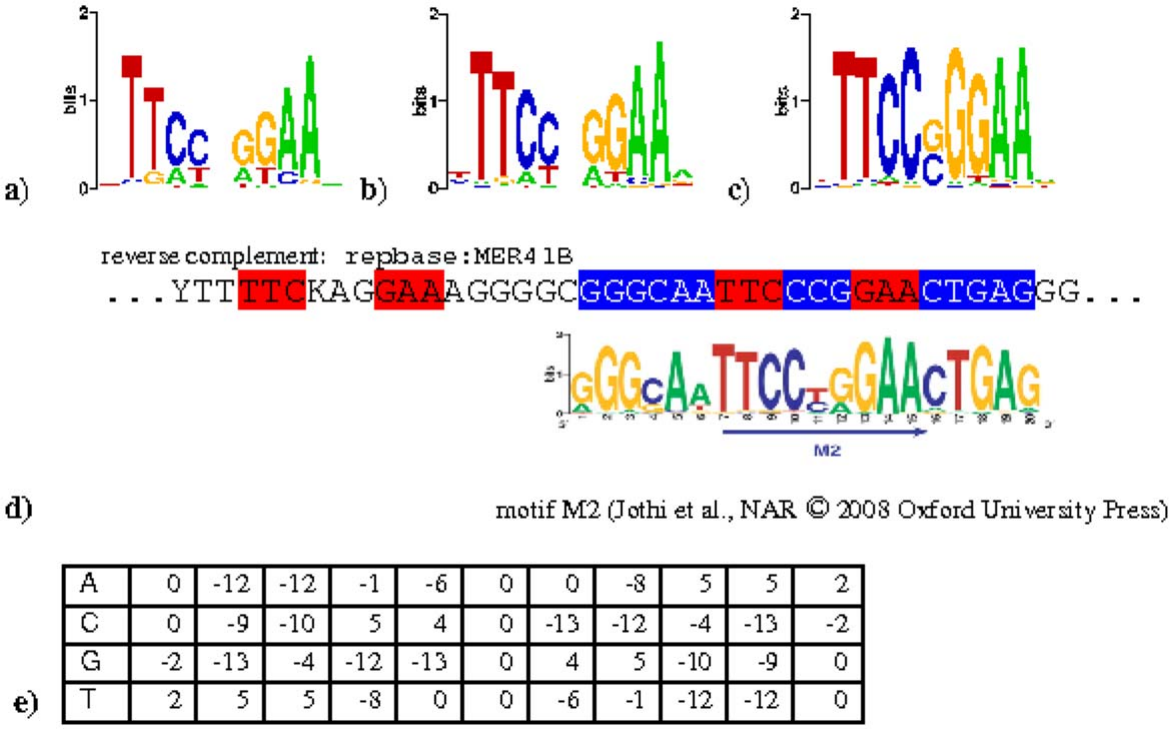
Descriptors of binding preferences of STAT1. Sequence logos (http://weblogo.berkeley.edu/) visualize the information content of occurrence frequencies of the 4 nucleotides by variable letter sizes at each position. Sequence logos of MAMOT motifs derived from in vivo ChIP-seq sites (a), repeat-filtered in vivo ChIP-seq (b), and in vitro SELEX sites (c). Note the symmetrical half sides with the presumable STAT1-interacting nucleotides are almost identical in a) to c). (d) Alignment of motif M2 (Jothi et al.) with the reverse complement of the consensus sequence of repetitive element MER41B (repbase). Table (e) specifies the PWM for STAT1 as visualized in panel a), each number indicating a score for matching nucleotides at corresponding positions.

We use the PWM (Fig. 2c) derived from in vitro SELEX assays [14] as initial model and apply a probabilistic modeling tool [24] for sequence motif discovery (results in Fig. 2a (sequence logo), and Fig. 2e (PWM)). Repetitive sequences with a considerable number of virtually identical sequences tend to impose repeat-specific characteristics of motifs in probabilistic approaches on repeat-containing sequence sets. To control for eventual biases brought in by repeats, we compare the results of independent motif discovery approaches on the repeat-containing sequence set of the 4446 STAT1 sites (see above) and on a subset of 3267 sites depleted of repetitive sequences. The almost identical motif resulting from motif discovery on a repeat-filtered set (Fig. 2a & b) indicates a minimal bias by repetitive sequences. The resistance to bias by repetitive sequences may however be intrinsic to the method of motif discovery. An independent reanalysis of the identical ChIP-seq data set using the popular motif discovery program MEME [25] on a very similar set of ‘high-coverage’ binding sequences [7] reports two motifs named M1 and M2. M1 is virtually identical to the motif found by us. M2 is a highly conserved 20mer sequence containing a classical GAS motif in the middle. Upon further analysis (see below) we find that this motif is nearly identical to a part of a repetitive sequence element containing two GAS sites (Fig. 2d). Based on these observations, we conclude that M2 is a motif discovery artifact reflecting repetitive sequences in the human genome rather than the intrinsic binding preference of STAT1.

A recent high-throughput study of transcription factor binding specificity challenges our molecular understanding of how proteins interact with their DNA binding sites by the conclusion that roughly half of the analyzed proteins recognize multiple distinct motifs [26]. In order to consider this finding, we re-analyzed our collections of highly enriched *in vivo* STAT1 site with an algorithm reporting multiple motifs (MEME). In this case, the results obtained with the complete and repeat-filtered sets are strikingly different. With the complete set, we found three motifs with E-values in the order of 10^−1000^ or lower (Table 1). The top-ranked corresponds to the GAS motif. The second and third closely resemble parts of repetitive element MER41B and alpha satellite DNA, respectively, reproducing a bias by repetitive sequence as observed in the results by Johti et al. With the repeat-filtered set we also found at the top of the list a motif containing a GAS site with very low E-value. The second and third-ranked motifs consist of homopolymers and have considerably higher E-values. Homopolymers are strongly over-represented in the human genome and therefore are, together with repetitive sequences, frequently picked up by some of the motif finding programs. The still very low E-values of the homopolymer motifs in the order of 10^−200^ can be explained by the fact that the background sequence model used by MEME does not account for their over-representation in natural DNA. In our interpretation, the lack of any additional motif ranking higher than commonly found homopolymer motifs constitutes evidence that IFN-γ induced STAT1 recognizes only a single motif in the non-repetitive part of the human genome. In conclusion the binding preferences of STAT1 in vivo are very comparable to the in vitro binding of recombinant STAT1 protein to random oligonucleotides. And the binding sites occupied by STAT1 in vivo upon IFN-γ stimulation do not diverge between repetitive or non-repetitive genomic loci.

**Table 1:** MEME reports motifs derived from repetitive sequences.

Motif number	Consensus sequence	E-value in MEME
all STAT1 sites		
M1	T**TTC**CCG**GAA**	**7.7e-3209**
M2	TCCACCCCTTGTTTAGCATATAATCA	**1.6e-1501**
M3	TGATGTGTGCATTCAACTCAC	**9.1e-848**
repeat-filtered		
M1	GAT**TTC**CGG**GAA**ATG	**2.0e-2351**
M2	AAAAAAAAAAAAAAAAAAAAA	**9.6e-205**
M3	CCCCTCCCCCGCCCCCCCCCC	**5.3e-202**

Motif discovery using MEME with default parameters on sequences spanning either the complete set or a repeat-filtered set of STAT1 sites derived from ChIP-seq data. Top 3 motifs (M1 to M3) for both cases are displayed by consensus sequences and the corresponding E-values indicating an estimate of the number of motifs with similar or better statistics in a random sequence set. The striking differences among the secondary motifs indicate a strong bias by repetitive sequences (M2 and M3 of the complete set resemble sequences from MER41B, and satellite Satellite/ALR/Alpha, respectively). Their absence in the repeat-filtered set furthermore suggests a single motif for STAT1 binding in the non-repetitive part of the human genome.

### Limited occupation of potential STAT1 sites in specific cell type

**Figure 3:**
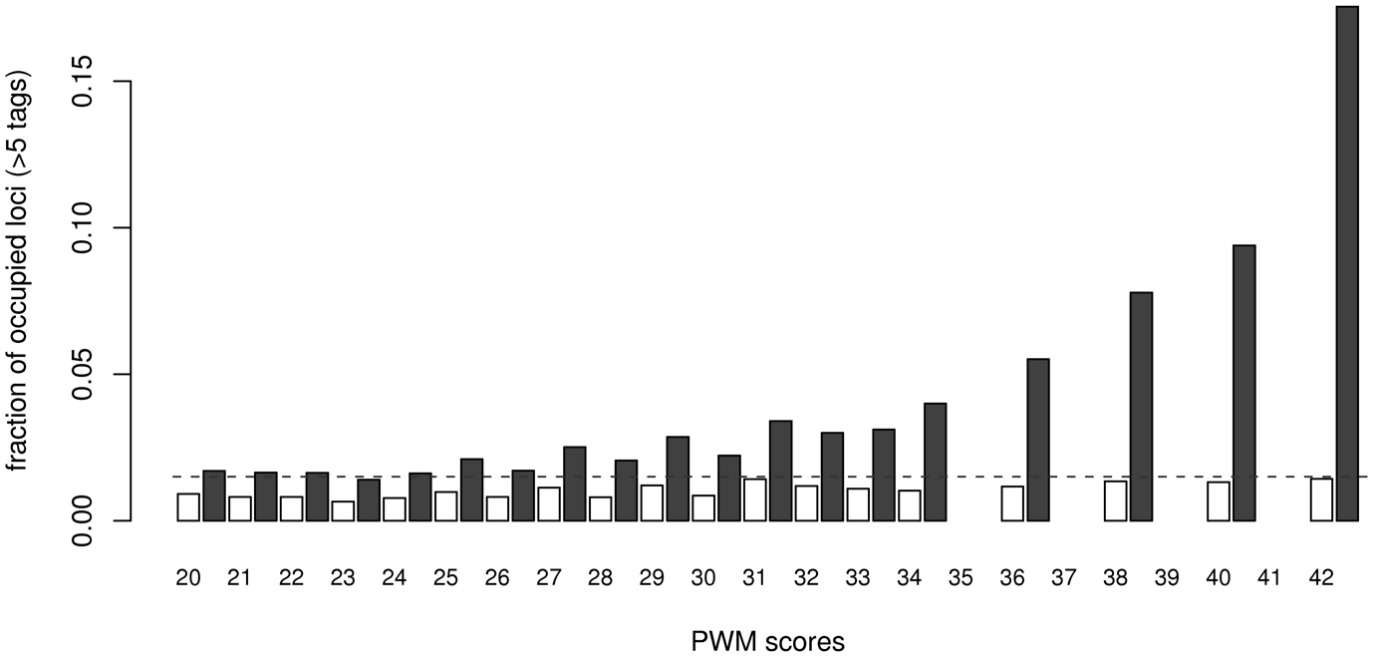
Estimation of PWM cutoff score from fraction of occupied STAT1 binding sites. 5 454 192 potential STAT1 binding sites in the human genome are grouped according their PWM score. In general the number of occurrences decrease with increasing PWM score, i.e. 664 981 sites for PWM 20, 213 877 for 30, and 7471 for 42. Missing bars are due to lack of combinations generating the corresponding PWM score. Occupation is defined as the fraction of sites covered by more than 5 ChIP fragments (within +/−100 bp). Plotted are data derived from ChIP-seq experiments with unstimulated (open boxes) or IFN-γ-stimulated HeLa cells (filled boxes). The dotted line represents the occupation frequency (0.015) at a collection of 4819 random genomic sites in stimulated cells.

As shown above, most loci with strong ChIP-seq signals are associated with an occurrence of the consensus STAT1 binding site. Therefore we address the question if the genomic nucleotide sequence and the refined PWM could be used to predict ChIP-seq tag counts. Our refined PWM allows to establish a comprehensive catalog of 5 454 192 potential STAT1 binding sites in the human genome, if using a deliberately low-stringency PWM score (> = 20, PWM in Fig. 2e). For each PWM score class we compute the fraction of binding sites occupied by more than 5 ChIP-seq tags within 100bp distance to the predicted binding site. This fraction reflects the in vivo occupation by STAT1, which clearly increases with higher PWM scores (Fig. 3). Thus for cells stimulated by IFN-γ, the better a genomic sequence is matching the PWM, the higher is the probability to be occupied by STAT1. Conversely, the fraction of occupied sites inferred from the ChIP-seq experiment with unstimulated cells, is largely independent of the PWM score. This finding indicates that in unstimulated cells, sequences with putatively higher affinity are not preferentially bound by STAT1 and sampled by ChIP-seq. Therefore the ChIP-seq signal in unstimulated HeLa cells consists to a large extent of unspecific background sequences [3].

Figure 3 suggests furthermore a PWM score of 30 as threshold for binding sites exhibiting a marked difference in occupation from background levels. Therefore such analysis based on in vivo occupation may complement statistical approaches to define PWM cutoff scores [27], [28].

While the observed higher occupation in the ChIP-seq experiment at sites with higher affinity is conceivable with models of DNA binding [29], the average occupation remains unexpectedly low. The fraction of occupied sites with PWM score above 30 remains below 4%, and even high affinity sites with the maximal PWM score are occupied at a frequency of less than 18%.

The specificity of the ChIP-seq signal is further underlined by the coverage at a collection of 37 experimentally characterized STAT1 binding sites [22] including both GAS and ISRE sites. 7 of these loci contain only ISRE motifs and lack any GAS matches (score >30) within 300bp to the ISRE motif. The ISRE motif [15] is mainly interacting with STAT1–STAT2–IRF9 complexes formed following stimulation with type-I-interferons, but not with IFN-γ [30]. Accordingly these ISRE loci are occupied by an average of only 18.1 ChIP-seq tags, whereas the remaining 30 loci containing GAS exhibit an average of 311.9 tags. The latter number represents a 60-fold excess of the threshold of 5 tags applied to determine occupation, evidencing sufficient ChIP-seq sequencing coverage for our approach. In summary the comparison of sequence-based prediction of STAT1 binding and of observed ChIP-seq tag counts indicates that the predictions are hampered by a high number of false positives. This may results in a reasonable sensitivity, but a very low specificity.

### Repetitive elements MER41 contain STAT1 binding sites

Approximately one forth of our STAT1 binding sites map within annotations of repeats (RepeatMasker track in UCSC, [31]). Remarkably the relative number of STAT1 ChIP-seq reads within repetitive sequences displays a strong increase following IFN-γ stimulation, ruling out potential systematic mapping artifacts related to repetitive sequences or to the completely abnormal karyotype of HeLa cells. The induction of the STAT1 binding is though restricted, the complete class of LTRs and also other repeat classes do not display significant changes in normalized numbers of ChIP-seq tags upon treatment with IFN-γ (Table 2). Conversely the medium reiteration frequency interspersed repeats MER41B of the class of Long Terminal Repeats (LTR) feature almost 20-fold more ChIP-seq tags in IFN-γ stimulated cells as compared to unstimulated control (log ratio 2.7 in Table 2). Accordingly 292 (6.5%) of our identified binding sites are located within MER41 annotations, collecting 41080 (5.4%) tags, while MER41 elements cover only approximately 4 Mb (0.1%) of the genome.

**Table 2:** Induction of STAT1 binding within specific repetitive sequences.

class	name	Ln ratio	stim	unstim	cov	space
LTR	MER41B	2.69	68619	3978	1069	21
*tandem GAS within reps*		1.97	65482	7819	1870	21
LTR	MER41E	1.65	3785	618	135	21
LTR	LTR19B	1.64	2460	407	116	+
LTR	MER93B	1.26	2540	616	134	+
LTR	LTR47A	1.19	4709	1222	228	49
LTR	MER41A	0.66	5834	2581	964	+
*tandem GAS outside rep*		0.63	32731	14948	2900	21
LTR	MER93a	0.60	2194	1026	192	+
LTR	LTR22C	0.59	1204	569	167	+
LTR	MER66C	0.54	1992	989	184	+
tRNA	Class	0.43	3541	1970	108	na
snRNA	Class	0.04	2410	1979	338	na
LTR	Class	0.03	1157617	955767	248357	na
SINE	Class	−0.07	1255938	1148004	388907	na

STAT1 ChIP-seq tag counts located within repetitive sequence annotations. The table is sorted according the strongest induction of STAT1 binding (column 3) and indicates in the top part all major repeat types which cover more than 100 kb of genomic sequence. The lower part shows major repeat classes in the genome annotation. For comparison the table includes also the STAT1 binding within 400 bp around the center of tandem matches to the STAT1 PWM either within or outside repetitive sequences (italic). Columns 4 and 5 indicate the number of centered tags within the corresponding repeat in the ChIP-seq data from IFN-γ stimulated cells or from unstimulated cells, respectively. The induction of STAT1 binding by IFN-γ is indicated by column 3 representing the natural logarithm of the ratio in (col. 4/col. 5) normalized by the total tag counts (stim: 15.1M; unstim: 12.9M). The 6th column indicates the genome coverage in kb and column 7 indicates the spacing in bp between tandem STAT1 sites within the corresponding consensus sequence. (+) indicates spacing larger than 100bp or a single STAT1 site. Only very specific repetitive elements display a significant induction, classes with highest coverage and tag counts (e.g. SINEs) display similar tag counts in IFN-γ stimulated or unstimulated cells. Tandem STAT1 sites inside repeat annotations display an almost 4-fold increased induction compared to similar sites outside repeats.

The specific induction as well as the comparable occupation of predicted binding sites inside repeats argues against a generalized ‘inactivation’ of repetitive elements as proposed earlier [32]. In support of the observed binding at MER41 elements, the consensus sequence of MER41B [33] contains two high scoring GAS arranged in tandem with a spacing of 21bp (center-to-center distance). Tandem GAS bound by hetero- or homotetramers of STAT family members have been reported previously with ambiguous spacings [34], [35], [36].

### Induced GAS tandems display a preference of spacing 18–21 bp

**Figure 4:**
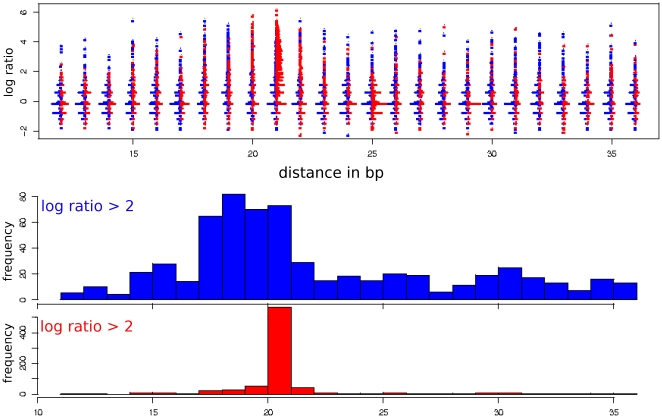
Spacings of GAS tandems differ in repetitive sequences. Putatively ‘high-affinity’ tandem GAS (average PWM score >30) are classified according the spacing between the centers of two sites (x axis), and the induction ratios (y axis). For each spacing class, histograms representing the frequencies of corresponding log ratios are displayed in vertical orientation. Red color indicates location within repetitive sequence annotations and blue specifies tandem GAS within non-repetitive sequences. Two histograms at the bottom summarize the data above for sites with induced binding (log ratio >2). Within non-repetitive sequences spacings 18–22 bp are moderately enriched in induced GAS tandems. For induced sites within repetitive sequences, a clear predominance of spacing 21 bp is observed, mostly related with MER41 repeats.

Eukaryotic transcription factors commonly act as multimeric complexes recognizing two or more DNA motif that occur at appropriate distances from each other. Tandem GAS sites may be part of such complex regulatory modules. Literature reports of tandem GAS sites in promoters of genes induced by STATs (for review [37]) support our observation of a coexistence of tandem STAT1 binding sites both within repeats as well as outside repetitive sequence annotations. In order to reveal features of tandem GAS sites potentially predicting their affinity to STAT1, we analyzed the occupation by ChIP-seq fragments at putatively ‘high-affinity’ tandem sites (average PWM score >30) depending on the center-to-center spacing between GAS tandem sites. The induction of STAT1 binding by IFN-γ stimulation and the spacing of GAS tandems exhibit considerable heterogeneity as displayed in Fig. 4, equally for sites within repetitive as well as within non-repetitive sequences. Concordantly with the limited occupation described above, a significant portion of sites exhibit no change in binding (log ratio = 0), mostly associated with 0 counts in both stimulated and unstimulated data sets. If selecting sites with induced binding (log ratio >2), repetitive sequences feature a high proportion of exact spacing of 21 bp, whereas sites within non-repetitive sequences display slightly less pronounced preferences for spacings of 18–21 bp (Fig. 4). Within repeats, 21 bp spacing is mostly associated with MER41 annotation.

In summary, occupied individual STAT1 binding sites within repetitive sequences do in general not differ significantly from similar sites in non-repetitive parts of the genome, as indicated by comparable populations of STAT1 binding sites observed in the refinement of PWMs. In contrast the distribution of spacings separating GAS tandems displays clear differences between repetitive and non-repetitive sequences. A combined genome-wide analysis would therefore lead to a biased value of 21 bp for an optimal spacing and incorrect conclusions on structural arrangements of STAT1 complexes on the DNA helix.

### Unoccupied STAT1 sites display phylogenetic conservation

Functional TFBS are plausibly under selective pressure and should thus display an enhanced conservation in closely related species. To assess the conservation of human STAT1 sites within non-repetitive sequences, PhastCons scores [38] were averaged over predicted high-affinity STAT1 sites with PWM scores >30. Repeat sequences spoil multiple genome alignments used for the computation of PhastCons scores, therefore we limit this analysis to 298 431 sites within non-repetitive sequences. We split the predicted STAT1 sites into 4 classes according to their occupation with ChIP-seq tags and further separate sites located at TSS (within 1 kb to annotated TSS) from those distant to TSS. Table 3 displays the statistics of number of sites and average PWM scores for each class. The average PWM scores only slightly increase with increased ChIP-seq occupation, likely due to limited occupation of predicted high-affinity STAT1 sites (Fig. 3).

**Table 3:** Predicted STAT1 binding sites classified according coverage by ChIP-seq tags in HeLa cells stimulated by IFN-γ.

Occupation class	1	2	3	4
# of ChIP-seq tags	0	1-5	6-14	>14
PWM score average (+/− standard deviation)	34.15+/−2.57	34.34+/−2.67	35.21+/−3.08	35.89+/−3.07
# of sites distant to TSS	109532	165577	9029	5050
# of sites at TSS	2821	4508	1001	913

298 431 high-affinity STAT1 sites (PWM scores >30) are split into 4 classes according to their occupation with ChIP-seq tags. The sets are further separated into sites located within 1kb to an annotated TSS and those distant to TSS.

**Figure 5:**
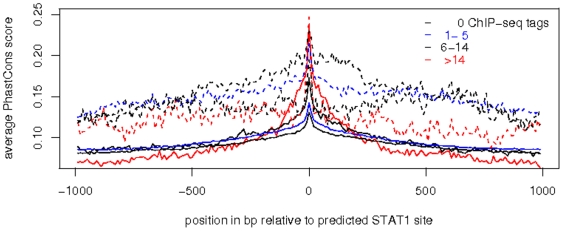
STAT1 sites unoccupied in HeLa cells nevertheless with increased phylogenetic conservation. STAT1 sites within non-repetitive sequences are classified according to the occupation by ChIP-seq tags and to their location either distant (>1 kb; solid lines) or close to annotations of TSS (dotted lines). For each of the sets (Table 3), the average PhastCons scores are computed at positions relative to the predicted STAT1 sites. In general STAT1 sites display a narrow increase of the average conservation score. (averages of PhastCons scores: genome wide 0.07; at TSS: 0.28). Closely neighboring TSS increase the average conservation, as well as higher ChIP-seq occupation tends to increased conservation at STAT1 sites. On the other hand TSS-associated STAT1 sites which lack any ChIP-seq tags still display a clearly augmented average conservation. This observation may suggest limited predictability of TF-binding in a specific cell type, even if information on nucleotide sequences with preferred binding (PWM) and on phylogenetic conservation are combined.

The resulting Fig. 5 shows a clear increase of the average of PhastCons scores precisely at the predicted positions for all sets of STAT1 sites. In general there is a tendency of increased conservation at STAT1 sites with higher ChIP-seq occupation, and sites close to TSS display a still increased and positionally broader conservation profile. The set of sites with highest occupation is associated with highest average conservation scores, remarkably independent on their location close or distant to annotated TSS. Striking is also the conservation profile of STAT1 sites close to TSS, but not occupied by any ChIP-seq tags in HeLa cells. The high average PhastCons scores of genomic sequences in close vicinity to the predicted STAT1 sites may originate from their location within regulatory modules. The clearly enhanced conservation of the precise location of STAT1 sites not occupied in HeLa cells might hint for a function most likely as binding site either in other cell types or by related members of the family of STAT transcription factors.

The sharp peaks with a width of a few 10 bp putatively identify conserved STAT1 sites, either isolated or within conserved regulatory modules. However does this analysis also detect STAT1 sites with augmented conservation which lack any ChIP-seq tags in stimulated HeLa cells. This suggests a limited predictability of TF-binding in a specific cell type, even if nucleotide sequences with preferred binding (via PWM) and phylogenetic conservation are combined.

## Discussion

### Aspects of ChIP-seq data analysis

This study aims at the identification of a reference set of robustly induced STAT1 binding sites upon IFN-γ stimulation. Many of the recent approaches in the analysis of ChIP-seq data use methods with similar underlying principles to determine genomic loci with elevated ChIP-seq signal (peak detection). Main differences consist in the determination of the threshold of signal intensities which separate signal from background noise. The noise level in ChIP-seq is still poorly characterized. Therefore the STAT1 binding motif obtained in the present study does not take into account putative low affinity sites. For the detection of low affinity binding sites, specialized approaches may be used [39], [40]. Approaches including also ‘weaker’ putative binding sites with very low ChIP-seq coverage did however so far not provide evidence for binding sites diverging significantly from the GAS motif [3], [12].

### Putative binding sites with very restricted occupation

The limited correlation of the predicted affinity (PWM score) and the observed STAT1 ChIP-seq signal as presented here suggests that genomic features additional to the nucleotide sequence determine the genomic binding of STAT1. Such mechanisms are likely generalized to all transcription factors and obvious candidates include cooperative effects between multiple DNA binding proteins. Epigenetic modifications may reduce the accessibility of the DNA by chromatin compaction [41] and nucleosome positioning might conceivably interfere with DNA binding [42]. In agreement with a number of recent studies [43], the prediction of DNA-protein interactions solely on the basis of the nucleotide sequence or on their phylogenetic conservation yields in an inaccurate set of sites actually occupied in a specific cellular condition. Unoccupied ‘perfect’ STAT1 binding sites may include cell type specific binding sites and suggest a required but not sufficient function of the nucleotide sequence for DNA-protein binding.

### STAT1 binding on repeats

The present study extends the analysis of STAT1 binding for the first time to repetitive elements. In distinction to previous reports on binding sites of other transcription factors within repeats [18], [19], [20], [21], we demonstrate a specific induction of STAT1 binding to selected repetitive sequence elements in reaction to a signal increasing the nuclear concentration of STAT1.

ChIP-seq signals are at present not reliably predictable by features of genomic sequences. Extended analysis of ChIP-seq data in HeLa cells including additional DNA binding proteins [12] and further sequence analysis approaches might however reveal combinations of features to better explain the observed binding of STAT1.

### Evolutionary aspects

The binding of STAT1 within repetitive sequences of the LTR class might relate to the description of functional GAS sites in retroviruses [44]. Of particular interest may be the fact that the detected MER41 repeats expanded only in the primate lineage. In contrast, the STAT1 pathway and thus STAT1 binding sites are found in species as distant as insects. Therefore the IFN-γ – STAT pathway precedes the expansion of MER41 repeats by several hundreds of millions years of evolution. Consequently the expansion of MER41 elements in the primate lineage likely remodeled parts of the pre-existing regulatory mechanisms of gene expression. This hypothesis is consistent with a previous study [18] concluding on an analogous role of a distantly related transcription factor (p53) binding to distinct LTR subfamilies. In particular MER41 may contribute to the divergence between primates and rodents. Thus the contribution of repetitive sequence elements could be included in detailed studies of the evolution of regulatory networks, exemplified by a recent analysis on the transcriptional repressor REST [45]. The DNA binding of TFs does however not allow definitive conclusions of transcriptional regulation of neighboring genes. Anticipating considerable experimental difficulties to target repetitive sequences by mutagenesis approaches, we discuss circumstantial evidence for potential functions of MER41 repeats in IFN-γ induced gene regulation. At first the presence of MER41 annotations in the 10 kb upstream regions appears not under negative selection pressure (Table 4). Unfortunately we could not find genome wide gene expression data of IFN-γ stimulated HeLa cells in public data repositories. At the example of the locus of SECTM1 on human chr17, we identified a gene regulated by IFN-γ in human monocytes [46] featuring an upstream MER41 element at 5 kb to the TSS with two high scoring GAS in tandem. A duplication event in the rodent lineage created two gene copies (Sectm1a and Sectm1b), which display clearly tissue expression patterns divergent from human SECTM1 [47]. Incidentally we do not find MER41 annotations in the upstream regions of a set of 10 genes with a common IFN-γ induction in both human and mouse tissues and associated with STAT1 binding sites [48]. Future comparative gene expression assays in corresponding cell types might focus on differential gene expression associated with the presence or absence of MER41 repeats in human and mouse, respectively.

**Table 4:** MER41 is under-represented in coding exons, but not in upstream regions.

	Number of MER41 instances	Length of genome compartment in Mb	Ratio over genome average
Genome	7 197	3 093	1
coding exons	8	35	0.09
10 kb upstream region	1 183	347	1.46

Annotations of MER41 repetitive sequence elements in the human genome (approx. average length: 330 bp) are selected depending on their location in coding exons or in 10 kb upstream regions of TSS annotations (at least 10 bp overlap). The cumulative length of the corresponding genomic sequences is used to determine a ratio relative to random expectation.

Our analysis confirms the previously described enhanced resolution of the ChIP-seq approach. However, ‘negative control’ data exploited by some of the current peak calling algorithms don't provide substantial advantages. We extend initial characterizations of STAT1 ChIP-seq data sets to binding sites within repetitive sequence elements. The selective induction of ChIP-seq signal at specific repeats upon cellular stimulation corroborates specific binding by STAT1. These observations bolster previous reports on binding sites of other TFs within repeats. Repetitive elements may however bring in biases deflecting the analysis of binding sequences. We recommend therefore an independent analysis of sequences derived from ChIP-seq data within repetitive and within non-repetitive genomic sequences, in order to avoid incorrect conclusions on general properties of binding sites.

## Materials and Methods

### ChIP-Seq data

The ChIP-seq data underlying this study are described in [3]. Results from ChIP-Seq experiments carried out with unstimulated and stimulated HeLa cells were downloaded from http://www.bcgsc.ca/downloads/chiptf/, providing 2 files with the genomic coordinates of 12.9 million, and 15.1 million mapped sequence tags of unstimulated and stimulated HeLa cells, respectively.

### Tag centering and peak detection

The source files were converted in SGA (Simple Genome Annotation) format, the working format of our ChIP-Seq tools available at http://ccg.vital-it.ch/chipseq/, http://sourceforge.net/projects/chip-seq/. An SGA file is a tab-delimited text file with five obligatory fields per line: chromosome, feature name, position, strand, and a number representing the count of sequenced tags mapping to this position. The position field corresponds to the chromosomal position of the 5′ end of the mapped sequence tag, that is the beginning of the matching region for tags on the positive (+) strand, or the end position of tags on the negative (−) strand. SGA files are sorted by chromosome, position and strand, allowing for rapid, sequential processing by downstream analysis tools.

The average length of the pulled-down fragments was estimated with the aid of the ChIP-cor program of the ChIP-seq tools. The ChIP-cor program generates a histogram indicating how many times a − strand tag is found at a particular distance of a + strand tag. In ChIP-seq experiments, + and − strand tags tend to occur in equivalent numbers in clusters around the transcription factor binding site. The relative displacement of + and − tags visualized by ChIP-cor serves as estimation of the typical fragment size, found to be 140 bp in these ChIP-seq experiments. An additional ChIP-center program generates “centered” SGA files by adding or subtracting a user-defined distance from the positions of the + and − tags, respectively, in the input SGA files. The strand field is changed to 0 to reflect the unoriented nature of the center positions defined in the output SGA file. Based on the estimated fragment length, we used a centering distance of 70 bp for SGA files of both stimulated and unstimulated ChIP-seq experiments.

The centered SGA files were used as input to the peak detection program ChIP-peak. The following summarizes the concepts of the ChIP-peak program. Each line of the sorted input SGA file is considered as candidate peak, for which the total number of mapped tags in a window of chromosomal positions is computed using neighboring lines in the SGA file. This window is centered on the position, with a user-specified width. In order to be retained as a peak, a candidate position must have at least a threshold number of total tags. Moreover, it must be the position with the highest number of counts within a so-called vicinity range, an additional user-specified parameter of the width of a position-centered window. If an input SGA line is retained as a peak, its position may be optionally redefined as the center of gravity of the tag counts in the surrounding window. The weight of sporadically occurring positions with suspiciously high tag counts can be decreased by a user-specified count cut-off value. Counts in the input file exceeding this value are replaced by the cut-off value. In this work, we used a window width of 200 bp, and a vicinity range of 400 bp, a (stringent) threshold of 50 counts, and a permissive count cut-off value of 999999. A posteriori peak refinement was turned off.

### GAS motif refinement

The refinement of the STAT1 binding motif description was carried out using the MArkow MOdeling Tool (MAMOT [24]) starting from the same initial Hidden Markov Model (HMM) as in a previous work [14]. As a training set, we used 200 bp long sequence fragments centered at 4446 peak positions obtained as described above. The STAT1 binding site model was refined with MAMOT implementing classical Baum-Welch training with the following parameter settings:
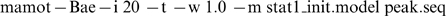



The initial model considers alternative spacer lengths of 2, 3 and 4 bases between the consensus half-site motifs TTC and GAA in the initial model. The resulting trained HMM assigned very low probabilities (below 1%) to the spacing classes 2 and 4, which allows to ignore these classes and to represent the STAT1 binding specificity by a standard position weight matrix (PWM) with spacer length 3. The probability matrix extracted from the trained HMM (shown as sequence Logo in Fig. 1a) was converted into an integer PWM (shown in Figure 2a) using the following formula:
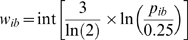
p_ib_ is the probability of base b at binding site position i, and w_ib_ is the corresponding weight in the scoring matrix. The function int rounds the argument to the nearest integer. The choice of the scaling factor 3/log(2) is arbitrary. Note that 3 score units correspond to a factor of 2 in terms of base frequencies. Putative binding sites can be scored by aligning their nucleotide sequence to the PWM and sum the matching scores over all positions.

### Determining the occupation of predicted sites

A genome-wide map of predicted STAT1 binding sites was generated as follows. A list of 11-mer sequences matching the weight matrix shown in Fig. 2a with a score ≥20 was compiled with a perl script. The fetchGWI program [49] was used to determine all exact matches of all corresponding 11-mer sequences on the genome. We then determined STAT1 occupancy by counting the number of centered tags from the stimulated and unstimulated data set within a window of ±100bp relative to the center position of predicted sites.

### Measure of induction of STAT1 binding by IFN-γ

The counts of the stimulated and the unstimulated ChIP-seq tag counts at a specific locus were augmented by one pseudocount, normalized by the total number of tags in the corresponding ChIP-seq experiment (stim: 15250744; unstim: 13019977 tags), and the natural logarithm was calculated of the ratio ‘stimulated’ over ‘unstimulated’ (log ratio).

### Average conservation scores at STAT1 binding sites

PhastCons scores on human genome coordinates derived from the 17-way vertebrate genomes alignment were obtained from the UCSC genome browser [31]. Converting PhastCons scores for each position into a density representation allows for efficient computation of average PhastCons scores using ChIP-cor (http://ccg.vital-it.ch/chipseq/) and applying count density normalization.

## Supporting Information

File S1 List of 4446 STAT1 binding sites in sga format (uploaded to ChIP-seq web server).
(0.14 MB TXT)

File S2 List of 4446 STAT1 binding sites in bed format (uploaded to UCSC Genome Browser).
(0.15 MB TXT)
